# Infectious healthcare waste management among private dental practitioners in the Rabat-Salé-Kénitra region, Morocco: A cross-sectional study on knowledge, attitudes, and practices

**DOI:** 10.25122/jml-2023-0038

**Published:** 2023-07

**Authors:** Lamiaa Abdallaoui Maan, Fatima Zahra Lachguer, Amal Bouziane

**Affiliations:** 1 Department of Periodontology, Faculty of Dental Medicine, Mohammed V University, Rabat, Morocco; 2 Private Practice, Khouribga, Morocco; 3 Laboratory of Biostatistics, Clinical Research, and Epidemiology, Mohammed V University, Rabat, Morocco

**Keywords:** infectious healthcare waste, waste management, dentists, Morocco

## Abstract

The increase in dental healthcare facilities and the use of single-use equipment have increased the production of healthcare waste. Their mismanagement exposes healthcare workers, waste managers, and the population to an infectious risk and negatively impacts the environment. Therefore, a correct management procedure has to be adopted from separation through storage to disposal. This study aimed to investigate dentists' knowledge, attitudes, and practices regarding managing infectious healthcare waste in private dental offices. A descriptive and analytical cross-sectional survey was conducted between December 2020 and March 2021 among private dentists registered at the Moroccan National Council of Dentists in the Rabat-Salé-Kénitra region. A questionnaire was developed to assess waste management in dental offices. Of the 500 questionnaires distributed, 190 completed and exploitable questionnaires were collected. Only 27.3% of healthcare waste managers in dental practices received training, 21,5% of practitioners assimilated the used gloves into household waste, 71.5% disposed of the waste generated by their offices directly into public bins, and 86.4% were unaware of Moroccan law 28-00 on waste management and disposal. This study highlights dentists' apparent lack of knowledge regarding healthcare waste management, and significant gaps were identified between actual practices and recommended regulations. To address these issues, developing a comprehensive medical waste management plan is crucial to encourage the practical cooperation of all stakeholders in this sector.

## INTRODUCTION

Nowadays, healthcare waste (HCW) is continuously increasing due to population growth, the development of healthcare facilities, and the increasing use of disposable medical products [1]. Similarly, with the onset of the COVID-19 pandemic, a fivefold increase in HCW generation has been recorded in many countries compared to the pre-Covid era [2].

According to the World Health Organization (WHO), 75% to 90% of waste is comparable to household waste and is not hazardous. The remaining 10-25% are considered dangerous, resulting in public health risks and catastrophic environmental impacts due to poor management [3].

Among the waste generated annually in Morocco, a significant portion consists of healthcare waste (HCW) or medical and pharmaceutical waste (MPW) from hospitals. It is estimated that hospitals in Morocco produce approximately 21,000 tons of HCW each year, with hazardous medical waste accounting for about 28% or 5,979 tons [4]. However, Morocco has pledged to continuously improve environmental protection in line with the national sustainable development strategy for several years.

**Figure 1:**
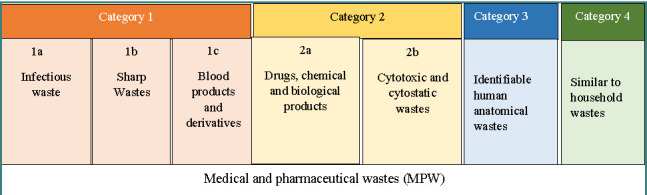
Summary diagram of MPW classification in Morocco according to Decree n°2-09-139 [33]

Recognizing the need for ecological and rational management of medical waste, the Moroccan government has implemented a range of strategies and legal frameworks to govern this sector, including Law No. 28-00 (2006) and Decree No. 2-09-139 (2009), on the management of MPW. According to articles 3 and 6 of this decree, MPW is classified into four categories based on their nature and characteristics, outlining specific procedures for sorting, packaging, storage, collection, transportation, treatment, and disposal (Figure 1) [5, 6].

However, most healthcare facilities dispose of their HCWs in an uncontrolled manner [4]. Furthermore, despite the increased number of private dentists practicing in Morocco and the similar increased production of different types of dental care waste, few studies have been carried out in private dental practices on managing the waste produced [7–9]. Most studies are epidemiological surveys or case studies at the level of large hospitals or public health facilities [10–18]. However, such studies are critical to establishing practices to allow health organizations to implement the necessary measures to correct the observed dysfunctions.

Therefore, the present study aimed to investigate the knowledge, attitudes, and practices (KAP) concerning managing HCWs at risk of infection among private dentists. This study excluded liquid, amalgam, chemical, and toxic radiological waste, subject to specific regulations.

## MATERIAL AND METHODS

This cross-sectional survey was carried out for descriptive and analytical purposes between December 2020 and March 2021.

### Sample

The study included practitioners registered with the Moroccan National Council of Dentists and worked in private dental offices or clinics in the Rabat-Salé-Kénitra region. Dentists who did not practice in the private sector, replacement dentists, or those who declined to participate were excluded from the study.

### Questionnaire and studied variables

A questionnaire was developed to assess the management of healthcare waste (HCW) generated by dental practices. The questionnaire was based on previous studies [9, 19–21] and supplemented to cover various aspects of HCW management. Content validity was determined through expert judgment, and the questionnaire was improved based on their feedback. It was distributed directly to practitioners in their offices and sent to their electronic addresses.

The questionnaire aimed to explore private practitioners' knowledge, attitudes, and practices related to HCW management. It included closed and open-ended questions divided into eight parts, covering socio-demographic characteristics, training in waste management, quantity and composition of waste generated, conditions of waste management from production to disposal, management of health risks, and general questions.

Specifically, items were used in the questionnaire to assess participants’ knowledge of household waste identification, compliance of waste conditioning equipment with international standards, treatment and disposal mode of contaminated waste, and healthcare risks inherent to HCW. Additional items were formulated to evaluate participants’ attitudes towards HCW management, such as reasons for not using a specific waste management disposal, awareness about Moroccan law 28.00 relating to waste management, satisfaction with their waste management system, and suggestions for improvement. The other items were designed to assess their practices regarding HCW management, from production to disposal and prevention of related healthcare risks. All questionnaire respondents were included in the study, and efforts were made to avoid duplicate or repeated responses to ensure data accuracy and reliability.

### Statistical Analysis

Cronbach's alpha coefficient was calculated to assess the reliability of the practice items in the questionnaire. An alpha coefficient >0.6 was considered acceptable for internal consistency. The internal consistency measurement in this study estimated Cronbach's alpha coefficient at 0.651.

The sample size was calculated based on the percentage of practitioners performing sharps waste separation in special containers. This percentage was estimated at 89.9% in the study by Brunot and Thompson [19]. The sample size was initially estimated to be 151 based on 0.05 first species error, 0.89 theoretical prevalence, and 0.05 accuracy. All private dentists (a total of 500) were invited to complete the questionnaire to cover the computed sample size.

Quantitative variables were reported as mean and standard deviation for symmetric distribution and median and quartiles for asymmetric distribution. Categorical variables were expressed as counts and percentages. An analysis was conducted to determine a possible association between different variables (such as training, sorting of sharp waste, and sorting of HCW) with waste management attitudes. The Chi-square or Fisher's Exact test was used to compare categorical variables. Statistical significance was set at a p-value of 5%. Data analysis and graphical representation were performed using Jamovi (statistical software, version 2.2) and Microsoft Excel (version 16.0).

## RESULTS

### Socio-demographic characteristics

A total of 190 private dentists completed the questionnaire, resulting in a response rate of 38%. The participating dentists were evenly distributed between men and women and represented three cities in the Rabat-Salé-Kénitra region. Most dentists (67.4%) practiced in Rabat, and most (96.2%) practiced in dental offices rather than clinics. Additionally, 38.4% of the participants had more than 15 years of clinical experience (Table 1).

**Table 1:** Socio-demographic characteristics of the practitioners surveyed

Characteristics*	Values (N=190)
**Gender** Female Male	94 (49,7%) 95 (50,3%)
**Age (Years) M ± SD**	41,5±10,3
**Practice duration (years)** <5 5 to 15 Ten to15 >15	42 (22,1%) 27 (14,2%) 48 (25,3%) 73 (38,4%)
**Practice place** Office Clinic	176 (96,2%) 7 (3,8%)
**City** Rabat Sale Kenitra	128 (67,4%) 43 (22,6%) 19 (10,0%)

M±SD: Mean ± standard deviation; *Number and percentage

### Training in HCW management

About half of the dentists (52.6%) had basic training in waste management, and the majority (92.1%) had assigned dental assistants to manage office waste. Only 27.3% of the waste managers were trained on this topic (Table 2).

**Table 2:** Characteristics related to the training of practitioners and HCW managers

Characteristics*	Values (N=190)
Basic training Continuous training No training	100 (52,6%) 19 (10%) 86 (45,3%)
**HCW managers** Dentist Dental assistant Secretary Cleaner	49 (25,8%) 175 (92,1%) 2 (1,1%) 36 (18,9%)
**HCW Manager training**	51 (27,3%)

* Number and percentage; HCW: Healthcare waste

### Type of waste generated and quantification

Dentists reported generating various types of HCW, but approximately half of the respondents (49.7%) indicated that they did not know the quantity of waste generated in their offices (Table 3).

**Table 3:** Characteristics of waste generation

Characteristics*	Values (N=190)
**Type of waste** Sharp waste Waste contaminated by blood or other biologic liquid Dental amalgams Human anatomical waste Drugs/ Unused products Others	185 (97,9%) 184 (97,4%) 48 (25,4%) 170 (89,8%) 99 (52,4%) 5 (2,7%)
**The average quantity of infectious HCW generated** < 5kg/month 5 kg /month< HCW <100 kg/week HCW > 100 kg/week I don’t know	55 (2,4%) 39 (20,9%) 0 93 (49,7%)

* Number and percentage; HCW: Healthcare waste

### Conditions for sorting and packaging of HCW

**Figure 2:**
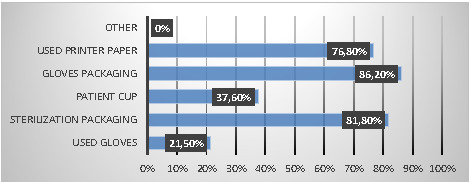
Respondents’ knowledge regarding HCW categories to be assimilated into household waste

Regarding the sorting and packaging of HCW, most dentists considered glove packaging, sterilization packaging, and used printer paper similar to household waste, with percentages of 86.2%, 81.8%, and 76.8%, respectively (Figure 2).

**Figure 3:**
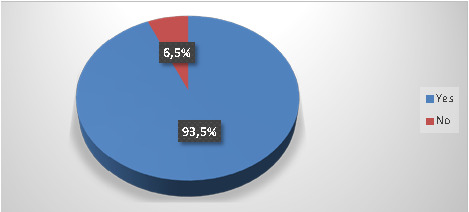
Disposal of sharp wastes separately from household wastes

**Figure 4:**
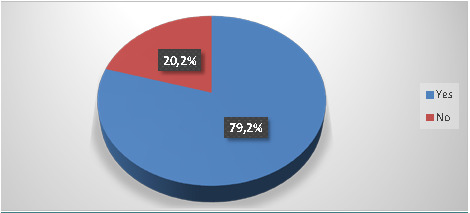
Disposal of infectious waste separately from household waste

For sharp wastes, 93.5% of dentists sorted them in bottles filled with plaster (53.2%) or specific rigid containers (39.8%) (Figure 3). Moreover, 79.2% sorted HCW separately from household waste (Figure 4). Only 12.2% opted for a color-coding system, and the majority (80.5%) reported filling packaging equipment to approximately 2/3 of its volume. Additionally, 58.9% of dentists were unaware of whether their equipment met international standards.

### Collection and storage conditions

Most participants (77.6%) had adequate waste containers for collection, and most (90.9%) disinfected them regularly. Approximately three-quarters (73.5%) reported having a specific storage area in their office, and a minority (7%) used the sterilization area as a storage place.

### Processing and disposal conditions

Only 25.1% of participants used a specific waste disposal route, with the majority (82.4%) collecting their waste from a service provider (Table 4). Conversely, 71.5% of dentists disposed of their waste directly in public garbage bins (Table 5). About half of the dentists who used a specific waste management disposal brought in or collected their waste once the bag was filled.

**Table 4:** Characteristics of waste treatment and disposal conditions

Characteristics *	Values
**Disposal of infectious HCW by specific route N=179** Yes No	45 (25,1%) 134 (74,9%)
**Methods of disposal N=51** Voluntary intake at an intermediate storage Voluntary intake to a service provider Collection at the office by a service company	6 (11,8%) 3 (5,9%) 42 (82,4%)
**Frequency of intake or collection N=148** Once a week Once a month Once every three months Every time the bag is filled Other	37 (25,0%) 30 (20,3%) 1 (0,7%) 74 (50%) 6 (4,1%)
**Existence of an agreement N=63** Yes No	8 (12,7%) 55 (87,3%)
**Existence of a traceability document N=67** Yes No	28 (41,8%) 39 (58,2%)
**Satisfaction with quality service N=60** Yes No	49 (81,7%) 11 (18,3%)
**Annual cost service N=71** Less than 1 000 MAD 1 000 to 3 000 MAD More than 3 000 MAD	29 (40,8%) 15 (21,1%) 27 (38,0%)
**Knowledge of infectious HCW treatment N=150** Yes No	20 (13,3%) 130 (86,7%)
**Infectious HCW treatment N=21** Incineration Disinfection Sanitary landfill	19 (90,5%) 5 (23,8%) 1 (4,8%)

* Number and percentage; HCW: Health care waste; MAD: Moroccan Dirhams

**Table 5:** Disposal of HCW apart from a specific route

Characteristics*	Values (N=130)
Dump directly into public bins Discharge into isolated areas Public bins/ Isolated area Other	93 (71,5%) 23 (17,7%) 9 (6,9%) 5 (3,8%)
**Reasons for not using a specific route for HCW disposal by dentists**	**Values (N=128)**
Not knowing the regulations Out of ignorance of the collecting societies Due to the lack of opportunity for close grouping Due to the cost Other	35 (27,3%) 67 (52,3%) 29 (22,7%) 53 (41,4%) 5 (3,9%)

* Number and percentage, HCW: healthcare waste

The annual cost of the services was estimated at less than 1000 MAD (Moroccan dirhams) per year for 40.8% of the practitioners. In addition, 13.3% were informed about the waste treatment method, of which 90.9% is incineration (Table 4).

The dentists who did not use a specific disposal method for their waste mentioned several reasons, the most important of which was the lack of knowledge about waste collection companies (52.3%) and the cost of services (41.4%) (Table 5).

### Prevention of health risks related to the management of HCW

In this study, 55.5% of dentists reported no history of injuries or cuts from sharp wastes. Nearly all participants used protective equipment such as gloves, masks, and gowns, while 46.9% used safety glasses. Most staff (95.1%) were aware of the health risks associated with HCW, but only 24.6% reported being fully vaccinated.

### Knowledge of the legislation and control of HCW management in dental practices

A small proportion of dentists (13.6%) stated they were sufficiently informed about Law 28-00 on waste management and disposal. Additionally, most dentists indicated that they had never been inspected by the Ministry of Health (97.5%) or the Municipal Hygiene Office (96.4%).

### Satisfaction with HCW management and proposals for improvement

When asked about suggestions for improving HCW management in their practices, dentists prioritized the need for information on waste collection in their geographical area (73.6%), training (63.2%), or contracting with a specialized company for biomedical waste treatment (61.5%). Other proposals included assigning responsibility to qualified personnel (36.8%), improving selective sorting at the office level (28.0%), and reducing waste at the source (13.7%).

### Relationship between basic training and dental practices and knowledge of HCW management

Among dentists with more than 15 years of practice, 56.7% had not received basic training in HCW management, compared to 22% who had received training (p<0.001).

A statistically significant difference was observed between dentists who received basic training and those who did not, in terms of sorting non-sharp HCW (p<0.025), methods of sorting, use of a specific waste disposal route (p<0.001), and knowledge of law 28-00 (p=0.014) (Table 6).

**Table 6:** Association between basic training and dental practitioners' practices and knowledge of HCW management

Variables	Basic training N=100	No basic training N=90	p
**Practice duration (years)** <5 5-10 10-15 >15	31 (31%) 20 (20%) 27 (27%) 22 (22%)	11 (12,2%) 7 (7,8%) 21 (23,3%) 51 (56,7%)	<,001
**Segregation of sharp HCW**	94 (95,9%)	78 (90,7%)	0,152
**Sorting method of sharp HCW** Household garbage A bottle filled with plaster Specific rigid container	6 (6,5%) 35 (37,6%) 52 (55,9%)	6 (7,7%) 56 (71,8%) 16 (20,5%)	<0,001
**Segregation of infectious HCW**	83 (85,6%)	62 (72,1%)	0,025
**Waste storage room**	72 (73,5%)	64 (73,6%)	0,988
**Specific waste disposal route**	35 (37,2%)	10 (11,8%)	<0,001
**The annual cost of services** Less than 1 000 MAD 1 000 to 3 000 MAD More than 3 000 MAD	17 (34,7%) 12 (24,5%) 20 (40,8%)	12 (54,5%) 3 (13,6%) 7 (31,8%)	0,323
**HCW disposal** Household garbage Isolated areas	41 (73,2%) 10 (17,9%)	52 (70,3%) 13 (17,6%)	0,951
**Knowledge of Law 28-00**	19 (19,4%)	6 (7%)	0,014

HCW: healthcare waste, MAD: Moroccan Dirham

### The link between HCW sorting and disposal and KAP of dentists on HCW management

Less than a third of dentists (30.2%) who sorted their HCWs used a specific waste disposal system compared to 2.9% who did not (p<0.001). A statistically significant difference in the percentage of the annual cost of services was also noted between dentists who sorted their HCWs and those who did not (p=0.017) (Table 7) and between those who used a specific waste disposal route and those who did not (p<0.001).

**Table 7:** Association between sorting of infectious HCWs by dentists and their practices and knowledge in HCW management

Variables	Segregation of infectious HCW N=145	No Segregation of infectious HCW N=38	p
**Years of practice (years)** <5 5-10 10-15 >15	27 (18,6%) 25 (17,2%) 41 (28,3%) 52 (35,9%)	11 (28,9%) 2 (5,3%) 7 (18,4%) 18 (47,4%)	<0,085
**Specific waste disposal route**	42 (30,2%)	1 (2,9%)	<0,001
**The annual cost of services** Less than 1 000 MAD 1 000 to 3 000 MAD More than 3 000 MAD	21 (35,6%) 11 (18,6%) 27 (45,8%)	6 (85,7%) 1 (14,3%) 0	0,017
**HCW disposal** Household garbage Isolated area	70 (70,7%) 20 (20,2%)	22 (75,9%) 3 (10,3%)	0,546
**Knowledge of Law 28-00**	20 (14,1%)	3 (8,3%)	0,577

HCW: healthcare waste; MAD: Moroccan Dirham

Moreover, 34.4% were aware of law 28-00 among those who used a specific waste disposal route, compared to 7.5% who did not use it (p<0.001). Similarly, 97.7% who used a specific waste disposal route were satisfied with their current management of HCW, compared to 48.1% who did not use it (p<0.001).

## DISCUSSION

The findings of this study provide an overview of the knowledge, attitudes, and practices of HCW management in private dental practices in the Rabat-Salé-Kénitra region. The results highlight insufficient knowledge among dentists and non-compliance with Moroccan and international regulations regarding the various stages of HCW management.

### Quantification of HCW generated

Almost half of the participants stated that they did not quantify their HCW. This percentage is significantly higher than reported in a French study of liberal health professionals in the Dordogne region, where only 14% of respondents were unaware of the quantity of HCW waste produced in their offices [22]. Estimating the quantity of waste generated and its composition enables the planning of appropriate waste management processes, in particular, to anticipate the need for collection, packaging, and treatment equipment, evaluate the cost, and respect the storage times indicated by law [23]. In contrast to hospital waste, the quantity of HCWs produced by dental practices is not widely published in the literature. The information on HCW generation in dental practices is often based on questionnaires filled out during surveys [9, 22, 24] or through physical measurements of waste production [23-26].

### Training in HCW management

While 62.6% of dentists in this study reported receiving waste management training, more emphasis on this topic in the curriculum and continuing education is needed to fill the knowledge gap. This percentage is higher than in a survey by Daou *et al*. in Lebanon, where only 41% of participants received training programs on medical waste management [27].

However, despite the relatively high percentage of dentists who received training, there is still a concern regarding the involvement of housekeepers and secretaries in HCW management without proper training. The study found that only 27.3% of those assigned to waste management in private dental offices had received training in waste management. It should be noted that in the study carried out by Manyani *et al*. in the Rabat-Salé-Kénitra region among dentists in both the public and private sectors, 52% of waste managers received training [9]. This difference in results for the same province highlights the attention the Moroccan Ministry of Health paid to the public sector, where many training and awareness-raising activities have been carried out for staff managing HCW.

The study also established a link between waste management compliance and training, as evidenced by comparing waste management variables between dentists who had received basic training in HCW management and those who had not (Table 7).

Training in HCW management can improve the knowledge and practices of all healthcare managers involved, as evidenced by an interventional study by Ozder *et al*. in 2013 [23]. A comparison of the scores obtained in the knowledge tests performed before and after the training courses showed an increase in the knowledge levels of all volunteers who received training on waste management. As a result, the number of malpractices in households and medical waste collection was reduced [28].

Other interventional studies showed that educational approaches improved the knowledge and practices of healthcare professionals concerning the management of HCW but still need to be revised to change risk behaviors [29, 30]. Thus, to maintain appropriate control and establish a culture of prevention of occupational risks, it would be necessary to strengthen the skills of health professionals through continuing education and periodic assessment of their knowledge and practices.

### Methods of waste sorting and packaging

The purpose of source separation is to direct each type of waste to an appropriate route, thus reducing health risks and costs by reducing the volume and quantities of hazardous waste to be treated and disposed of [31].

This study showed that 79.2% of dentists sorted their infectious waste separately from household waste. These data align with those recovered in other studies on HCW management practices, including one conducted in Karnataka [20] and another study in Jeddah [32], where 70.5% and 73.2% of dentists, respectively, separated medical waste from household waste at source. However, although encouraging, our results concerning waste separation results must be considered cautiously because a significant percentage of dentists considered patient cups and used gloves similar to household waste (37.6% and 21.5%, respectively). The latter should be regarded as a waste at risk of infection because they “contain viable microorganisms or toxins that may cause disease in humans or other living organisms” [6]. This finding of incorrect sorting at the source transforms all wastes into infectious risk waste, with severe health and environmental consequences [16].

Furthermore, the selective sorting of medical waste requires using appropriate containers for each type. According to the Moroccan law (article 6 of decree n°2-09-139 of May 21, 2009), the waste is, as soon as it is generated, sorted according to its categories and put in plastic bags or in different colored single-use containers that meet the standards in force: red for category 1-a and 1-c waste; brown for category 2 waste; white non-transparent for category 3 waste and black for category 4 waste. Solid, hermetically sealed, yellow containers are also used for packaging category 1-b waste (sharp waste) [6].

According to the present study, only 12.2% of practitioners used plastic bags to sort and package different types of HCW. This finding aligns with a study by Singh *et al*. on 200 private dentists in India, who showed that 15.6% of practitioners used color-coded bags for packaging HCWs [32]. The figures seem better in a study in Jeddah. Among 314 dentists practicing in four dental colleges and 20 private dental clinics, 66.6% used color coding for plastic bags for packaging HCWs [33].

Moreover, 80.5% of our study’s participants performed the correct filling of the packaging equipment to 2/3 of the volume, but only 25% stated that the equipment complies with international standards. This finding aligns with another Moroccan study conducted by Mbarki *et al*. in seven hospitals in the Souss-Massa-Drâa province, which showed that only four hospitals (57.1%) had specific bags for collecting medical waste. However, most did not comply with international requirements such as puncture resistance, nor were they appropriately labeled [16]. These breaches of regulations can affect staff and the environment if the bags are punctured and their infectious and hazardous contents escape. Similarly, the lack of appropriate labeling indicating the nature of the waste makes it difficult for the public and collection staff to identify the source and type of medical waste [34].

The findings of the study revealed that there is a discrepancy in the management of sharps waste compared to non-sharp healthcare waste (HCW) among dentists. This observation is consistent with similar studies conducted in various African countries where dental wastes, excluding needles and sharps, are often mixed with household waste during collection and disposal [35]. Some dentists who reported sorting sharps waste in their offices used specific sharp waste containers (39.8%), and others used plastic bottles filled with plaster (53.2%). This practice remains a temporary solution that increases the weight and volume of waste. In addition, current Moroccan and international regulations require that sharp HCWs be sorted into rigid, leak-proof, red or yellow, puncture-resistant containers bearing the pictogram of infectious waste and equipped with temporary closure devices during use and permanent closure before removal [3, 6].

Moreover, training in HCW management positively impacts the sorting of sharp HCWs. Indeed, there was a statistically significant difference in using specific rigid containers for sorting sharp HCWs between dentists who had received training in waste management (55.9%) and dentists who had not (20.5%).

### Storage conditions for HCW

Articles 8 and 9 of the decree's third chapter emphasize an appropriate storage area far from the units generating medical and pharmaceutical waste and accessible only to specialized or operating staff [6]. In the present study, 86.7% of the practitioners stated that they had a specific storage area, and 7% used the sterilization area as a storage place. Although low, this proportion reflects a dangerous and unsafe practice. This discrepancy highlights the possibility of a misinterpretation by the practitioners regarding the term “specific” attributed to the storage area.

According to a survey in France, only 26.2% declared that they stored their HCWs in a dedicated room. This proportion rose to 35.5% among large producers [22] and 43.8% among producers of more than 5 kg in another cross-sectional survey [19].

### Conditions for treatment and disposal of HCW

It was observed that 71.5% of dentists disposed of their waste directly into public garbage bins, which is alarming considering the potential risks associated with improper disposal. This percentage is comparable to a survey conducted in Shiraz, Iran, where 89.1% of dental practices and clinics disposed of their waste and household waste, further highlighting the need for improved waste management practices [36].

Among the dentists who used a specific waste disposal route, which accounted for 25.1% of the participants, the majority (82.4%) opted for collection by a service company. These figures are still much lower than those of a study conducted in Latur city in India, among 82 private dentists practicing, where 87.5% of practitioners used a private service to collect biomedical waste from their clinics [37].

According to the legislation, an agreement must be made between the waste producer and a service provider responsible for container collection, transport, and disposal. According to the Moroccan Department of Environment and Energy, six private companies treat medical and pharmaceutical waste (MPW) nationally (e.g., Tozone Dasri in Témara). They provide services to health facilities at prices ranging from 7 to 11 MAD/Kg depending on the quantity and type of waste and transport costs ranging from 0.75 to 2 MAD/Kg [4]. This information needs to be sufficiently disseminated to the producers of HCWs. In our survey, the dentists who reported not using a specific disposal route for their waste mentioned several reasons, the most important of which was a need for more knowledge of collection companies (52.3%) and the cost of services (41.4%). This may also explain the large discrepancy between the percentage of dentists who reported sorting their waste (79.2%) and those who disposed of their HCW through a specific route (25.1%).

### Knowledge of Moroccan regulations and enforcement

The findings of this study emphasize the need for increased awareness of Law No. 28-00 on waste management and disposal. It is concerning that only 86.4% of the participants were aware of this law. Furthermore, it is noteworthy that the majority of the participants had never been subject to control by the Ministry of Health or the Municipal Hygiene Office (97.5% and 96.4%, respectively). However, these two organizations must ensure the respect of good practices and attitudes in managing medical and pharmaceutical waste, mainly since articles 70-79 of Law 28-00 apply several sanctions for regulatory violations [34].

### Protection against health risks related to HCW

All employees involved in the different stages of healthcare waste chain disposal, from production to final disposal, are at risk of exposure to infectious pathogens in healthcare waste. Pathogenic microorganisms can be transmitted through various routes, including direct contact, mucocutaneous exposure, aerosolization, or biological vectors [3]. Therefore, universal precautions are crucial, and healthcare workers should use protective barriers such as gloves, gowns, masks, and goggles to minimize the risk of exposure [38].

Our survey found that almost all practitioners used medical gloves, masks, and gowns when handling healthcare waste, which is a positive and satisfactory finding. However, only 46.9% of the participants reported the use of protective glasses. This figure is considered insufficient in dentistry, considering the systematic risk of splashing blood or other body fluids during dental procedures.

It is important to note that even with personal protective equipment, there is still a risk of cuts or injuries from healthcare waste, especially in accidental mishandling. 44.5% of participants had a history of cuts or injuries from waste during their practice. This prevalence is higher than the findings of a study conducted in Queensland, where the rate of blood exposure accidents among Australian dentists was 27.7% in the previous 12 months [39].

Needle stick injuries may facilitate the transmission of bloodborne pathogens such as human immunodeficiency virus (HIV), hepatitis C virus (HCV), and hepatitis B virus (HBV) [40], which explains the need for appropriate vaccination protection of personnel handling HCWs, including hepatitis A and B, as well as tetanus [38]. However, in our study, only 24.6% of waste management staff were vaccinated, which was insufficient to ensure their protection.

### Strengths and limitations of the study

The present study employed a representative sample of private dentists in the Rabat-Salé-Kénitra region, ensuring the generalizability of the findings within the specific geographical area. The sample size was calculated, contrary to Manyani *et al*. study, where only 50 completed and usable questionnaires were retrieved [9]. In our study, all dentists in the Rabat-Salé-Kénitra region registered at the dentists' northern regional council database were invited to respond to the questionnaire. Despite the relatively low response rate (38%), the number of participants was higher than the calculated sample size. Furthermore, the response rate was higher than that obtained in Manyani *et al*. study (29%) [9]. In general, response rates to surveys of HCW management practices are low [19, 22]. This raises questions about dentists prioritizing important health and safety issues [9]. Nevertheless, the results of our survey must be considered with caution, given the self-administered questionnaire, which may lead to biases in understanding and under-reporting, particularly for dentists with poor HCW management practices.

## CONCLUSION

HCW management practices in private dental practices in the Rabat-Salé-Kénitra region should be improved. Several actions must be implemented to ensure their improvement and compliance with recognized standards. All dental health professionals, including those in private practices, must be integrated into awareness programs, continuing education, and assessment processes to manage HCW. National guidelines should be widely disseminated to all healthcare facilities along with all regulatory documents defining roles, responsibilities, obligations, and penalties related to the improper management of HCW. Adequate monitoring teams with warning and sanctions powers must be organized to compel all generators of HCW to comply with the regulations in force. The ultimate goal is a medical waste management system that is harmonious with sustainable development and protects the environment and human health.

## Data Availability

The authors confirm that the data supporting the findings of this study are available upon request from the corresponding author.
